# An Unpleasant Though Interesting Experience

**Published:** 1896-03

**Authors:** Jno. E. Brown

**Affiliations:** Oskaloosa, Ia.


### REPORTS OF CASES.

### AN UNPLEASANT THOUGH INTERESTING EXPERIENCE.

BY JNO. E. BROWN, V.S.,
OSKALOOSA, IA.

MANY horses in this section of Iowa, during the past few months, have suffered with a disease somewhat similar to that described by Williams under the title of "Stomatitis Pustulosa Contagiosa." This is an exceedingly elaborate and generous name—sounds wonderfully scientific; but I shall take no exception, for, since my experience with the disease, I am prepared to indorse any sort of a "handle" that can be spiked on and made to stick.

The first case I saw was a standard-bred mare that had been sent out to pasture for a few weeks. On being notified that the mare was not doing well, the owner had her brought back to the city. By this time she could neither eat nor drink, and an empiric was called in to prescribe. After a critical (?) examination, he concluded that she must have an ulcerated tooth; but, not being able to find it, later diagnosed it "broken jaw," and said it was incurable.

At this stage of the play the owner telephoned to me that he had a mare with a broken jaw, and for me to bring such instruments and appliances as such a case required.

On arriving at his stable a most dejected and forlorn-looking specimen of the equine race was found. Like Mark Twain's donkey, she had to lean up against the side of the stall to think. Had taken no food or water for two or three days; was greatly emaciated and so weak that if made to move would almost fall down. Pulse weak and irregular, and the nervous system completely collapsed. Spasmodic twitching of the muscles of the whole body, but especially about the head and face. The involuntary action or jerking of the muscles of the masseteric region kept the teeth chattering together in a peculiar manner. The eyelids were almost closed, angles of the mouth drawn back, and the alæ of the nose distended. Owing to the muscular contraction, I could not get the mouth open. I tried to draw the tongue out through the interdental space, but it was too much swollen; was of a grayish color, with deep transverse furrows, and where pressure was brought to bear with my thumb and fingers in the attempt to draw it out, several small cracks opened up and blood oozed out. The mouth and the little saliva that dribbled away had an exceedingly offensive odor. There were no ulcers, blisters, or pustules outside of the mouth, but the Schneiderian membrane was much inflamed, and more or less of the secretions from the nose, which were scanty, adhered to the nasal openings.

I failed to find the "ulcerated tooth" or broken jaw. I could not compare the existing conditions with anything I had ever seen, except that of the epizoötic aphtha, which was so prevalent among the cattle of this country some years ago. The appearance of the tongue, mouth, lips, and mucous membrane, the great debility following, with other symptoms, were almost identical in the average case, and I cannot help associating the two diseases very closely together, if they are not the same.

The case described was the worst one I saw; the symptoms in the other cases being more or less modified. Neither did the disease appear as a contagion; in fact, it was the exception to find more than one or two cases in the same lot of horses. Generally it was the grazing horses that were attacked, though stable-kept horses were not exempt.

The principal treatment has consisted of frequent and thorough irrigation of the throat and mouth with a solution of hyposulphite of soda, and meeting urgent symptoms with such agents as seemed to be indicated.

In the cases where deglutition was difficult, a rubber tube was passed up through the nostril and the irrigation effected with a fountain syringe, and when it became necessary to administer stimulants or medicines by way of the stomach, the tube was pushed still further back and into the œsophagus. This line of treatment was most efficacious in every instance.

Particularly interesting to me, however, was my own individual case. On December 7th a man came to the hospital and said his horse had an ulcerated tooth which he wanted pulled. In this case the ulcerated-tooth theory was taken at par value and its location easily determined by the bulging of the superior maxillary directly over it, but no other abnormal conditions were apparent. After the tooth was extracted, which was done with the forceps, I hurriedly examined my hands, and, finding no abrasions or scratches, omitted my usual precaution of using an antiseptic wash, and went ahead with other business.

A few days later I began to feel somewhat indisposed; was semi-conscious of pain in the end of my left thumb; a small ulcer appeared on my left arm; also one on my right thumb. By the end of a week I began to fully comprehend that there was something the matter, and proceeded to investigate. All the above symptoms were becoming intensified, and coupled with chills, fever, and an inflamed condition of lymphatics extending from the ulcers to a painful swelling under my arms. Of course, it was then a clear case of septic poison, but where or when I got it remained a question for three or four days. Then the owner of the horse from which the tooth had been pulled came in with the evident intention of figuring conspicuously in arranging my epitaph, and found me nearer ready for an epitaph than he had anticipated. He said that a few days after the tooth had been pulled the mouth broke out with pimples and blisters, and he thought it had been poisoned by my instruments. From his description I readily recognized the disease above referred to, which must have been under incubation and sufficiently advanced for me to become inoculated either by the blood or saliva, and yet *not* sufficiently developed to attract attention. It later developed, also, that the inoculation had taken place at the corner of my thumb-nail, which had most likely been broken back to that extent by which the germ could find a lodgement and be introduced into the circulation, and yet the injury so slight that it was entirely unnoticed.

About the time the more aggravated symptoms of the poison began to subside I was taken with what would ordinarily be considered a bad case of bronchitis, and during its course almost all the symptoms seen in the horse were present to a greater or lesser degree.

I have been able to sit up now most of the time for nearly a week, but the prostrated and anæmic condition which a five-weeks' tussle with the disease and its complications left me in makes convalescence a slow business. During this time, however, I have had ample opportunity to consider various methods for the annihilation of animal life and carcass, a practical demonstration of which will certainly be given should I ever be permitted a chance to satisfy my revengeful nature on that horse.

